# Virtual Insights into Natural Compounds as Potential 5α-Reductase Type II Inhibitors: A Structure-Based Screening and Molecular Dynamics Simulation Study

**DOI:** 10.3390/life13112152

**Published:** 2023-11-01

**Authors:** Sibhghatulla Shaikh, Shahid Ali, Jeong Ho Lim, Khurshid Ahmad, Ki Soo Han, Eun Ju Lee, Inho Choi

**Affiliations:** 1 Department of Medical Biotechnology, Yeungnam University, Gyeongsan 38541, Republic of Korea; sibhghat.88@gmail.com (S.S.); ali.ali.md111@gmail.com (S.A.); lim2249@naver.com (J.H.L.); ahmadkhursheed2008@gmail.com (K.A.); gorapadoc0315@hanmail.net (E.J.L.); 2 Research Institute of Cell Culture, Yeungnam University, Gyeongsan 38541, Republic of Korea; 3 Neo Cremar Co., Ltd., Seoul 05702, Republic of Korea; melong-h@cremar.co.kr

**Keywords:** androgenic alopecia, androgen, dihydrotestosterone, 5α-reductase type II, natural compounds

## Abstract

Androgenic alopecia (AGA) is a dermatological disease with psychosocial consequences for those who experience hair loss. AGA is linked to an increase in androgen levels caused by an excess of dihydrotestosterone in blood capillaries produced from testosterone by 5α-reductase type II (5αR2), which is expressed in scalp hair follicles; 5αR2 activity and dihydrotestosterone levels are elevated in balding scalps. The diverse health benefits of flavonoids have been widely reported in epidemiological studies, and research interest continues to increase. In this study, a virtual screening approach was used to identify compounds that interact with active site residues of 5αR2 by screening a library containing 241 flavonoid compounds. Here, we report two potent flavonoid compounds, eriocitrin and silymarin, that interacted strongly with 5αR2, with binding energies of −12.1 and −11.7 kcal/mol, respectively, which were more significant than those of the control, finasteride (−11.2 kcal/mol). Molecular dynamic simulations (200 ns) were used to optimize the interactions between compounds and 5αR2 and revealed that the interaction of eriocitrin and silymarin with 5αR2 was stable. The study shows that eriocitrin and silymarin provide developmental bases for novel 5αR2 inhibitors for the management of AGA.

## 1. Introduction

Androgenic alopecia (AGA), also known as male pattern baldness, is a common type of hair loss. Hair is an essential bodily structure that protects the scalp, enhances human individuality, and serves a variety of purposes such as insulation, attractiveness, and tangibility [1]. AGA is caused by an androgen hormone imbalance, stress, hereditary diseases, malnourishment, 5α-reductase type II (5αR2) overactivity, thyroid dysfunction, drug addiction, and aging [2]. The testicles mainly produce androgens in the form of testosterone, which is then converted to dihydrotestosterone (DHT) by 5αR2. DHT interacts with androgen receptors in vulnerable scalp-based hair follicles and activates genes responsible for follicular shrinkage, thus causing AGA [3]. The absence of AGA in men with congenital impairment of 5αR2 demonstrates the relevance of DHT as an etiologic factor of this disorder [4]. Individuals with a genetic deficiency in the 5αR2 enzyme do not develop AGA. Furthermore, 5αR2 has been found in hair follicles on the scalp, with balding scalps exhibiting elevated levels of both 5αR2 activity and DHT concentrations. Overall, these findings support the rational use of inhibitors targeting 5αR2 as a therapeutic approach for treating AGA in men [5].

For many years, the treatment of AGA has been a point of contention in clinical dermatology. Many treatments for baldness are now available, including bioengineered hair transplants [6], hair follicle regeneration using rearranged stem cells [7], and medicinal treatment with the synthetic drugs minoxidil or finasteride [8,9]. Minoxidil stimulates hair growth by shortening the telogen phase and accelerating the transition to the anagen phase. It has also been demonstrated to expand hair follicles. In the treatment of androgenetic alopecia, minoxidil is still a significant advancement [10]. Finasteride is commonly used to treat androgen-dependent hair diseases such as androgenetic alopecia. This medicine is an orally given selective 5-alpha-reductase inhibitor used to treat androgenetic alopecia [11]. However, these drugs have been reported to have severe dermatological side effects, which include reduced libido, irritation, itching, erythema, and depression [12]. Hence, there is a pressing need for the development of a novel pharmaceutical agent that effectively stimulates hair growth while minimizing any potential adverse effects.

New drug development is one of the most important tasks of biomedical research from scientific and economic perspectives. However, despite the advancements made in informatics and computational biology, as well as parallel increases in drug development productivity, drug development has been sluggish due to heavy dependence on synthetic small molecules as a source of innovation. Computer-Aided Drug Design (CADD) has emerged as a highly effective technique for identifying promising lead compounds and advancing prospective pharmaceutical medicines addressing a wide range of disorders [13,14,15]. Currently, a variety of computational techniques are being used to identify interesting lead entities from large compound repositories. The application of CADD techniques in the context of drug development is progressing steadily. A prevalent trend in modern drug design revolves around the rational engineering of effective treatments with multi-targeting properties, improved efficacies, and reduced side effects, particularly with regard to toxicity considerations [16]. Natural products are a huge, diversified source of bioactive substances, and some have been utilized in traditional medicine for hundreds of years, which distinguishes them from synthetic small molecules [17]. The numerous health benefits of flavonoids, as described in epidemiological studies, have attracted the attention of the scientific community. These compounds are abundant in nature, particularly in fruits and vegetables, and possess diverse physical, chemical, and physiological properties. Several flavonoids have been well studied for their medicinal effects, which include antibacterial, hepatoprotective, antioxidant, anti-inflammatory, enzymatic activity modulation, anticancer, and antiviral activities [18]. Here, we aim to identify novel natural 5αR2 inhibitors using a computational approach, with the objective of finding a potential treatment for AGA.

## 2. Materials and Methods

### 2.1. Preparation of 3D Structure of 5αR2

The crystal structure of 5αR2 in complex with finasteride (PDB ID: 7BW1; resolution: 2.80 Å) was obtained from a protein data bank [19]. After removing water molecules and heteroatoms, including the co-crystallized ligand (finasteride), the clean structure of 5αR2 was prepared using the ‘prepare protein’ tool of the Discovery Studio 2021 (DS) and saved in monomer and .pdb format.

### 2.2. Retrieval and Preparation of Flavonoid Library

The unique collection of 241 flavonoid compounds was obtained from Selleck Chemicals (https://www.selleckchem.com (accessed on 13 December 2022)) in .sdf format. These compounds were minimized, prepared with DS, and converted to ‘pdbqt’ format for docking analysis.

### 2.3. Structure-Based Virtual Screening

The preeminent methodology used for the discovery of novel lead compounds in the field of drug development involves the physical evaluation of large chemical libraries against a specific biological target, also known as high-throughput screening. A complementary strategy, known as virtual screening (VS), entails the computational evaluation of large chemical repositories to identify molecules that exhibit complementarity to structurally characterized targets; compounds predicted to exhibit favorable binding characteristics are then subjected to experimental validation [20,21]. Receptor-based methodologies, also known as structure-based techniques, are intended to elucidate the interaction dynamics between a ligand and its receptor. Their primary goal is to distinguish between ligands with strong affinities for the target protein and those with weaker affinities. The possession of a three-dimensional target configuration is a critical requirement for carrying out a receptor-based VS initiative. This can take the form of a crystalline X-ray structure, an NMR-derived structure, or even a structure inferred through homology modeling. The dominance of receptor-based methodologies over ligand-based methodologies is growing, owing to the increasing availability of resolved three-dimensional structures of target proteins for research purposes. Structure-based VS anticipates the location and orientation of a ligand when it interacts with a protein [22]. In the present study, AutoDock Vina 1.1.2 [23] and AutoDock 4.2.5.1 [24] were used for VS and molecular docking studies to identify binding conformations with the lowest binding energies (BEs). Finasteride was used as a positive control for VS, and X, Y, and Z coordinates were set at −20.40, 17.50, and 45.54, respectively.

### 2.4. Physiochemical and Toxicity Prediction

The SwissADME web server was used to assess the predicted physiochemical and pharmacokinetic properties of the top two compounds (eriocitrin and silymarin) [25], and ProTox-II, an open-access web server, was used to predict their toxicities [26]. ProTox-II combines molecular similarity, pharmacophore, and machine learning models to predict toxicity endpoints, such as hepatotoxicity, immunotoxicity, carcinogenicity, mutagenicity, and cytotoxicity.

### 2.5. Molecular Dynamics (MD) Simulations

Studying the intramolecular dynamics of proteins can reveal hidden biological functions and intricate mechanisms. GROMACS2019.6 [27] was used to study the stabilities of 5αR2-eriocitrin, 5αR2-silymarin, and 5αR2-finasteride (control) complexes. MD simulation was carried out for 200 nanoseconds (ns) using the GROMOS96 43a1 force-field parameter [28]. The protein topology file was generated using the gmx tool, while the ligand topology file was created using the Swiss Param server. Subsequently, the TIP3 water model was employed, and a solvent box was generated at a distance of 10 Å. To achieve system equilibrium, counter ions such as Na^+^ and Cl^−^ (5αR2-eriocitrin; Na^+^ (0), Cl^−^ (10), 5αR2-silymarin; Na^+^ (0), Cl^−^ (10), and 5αR2-finasteride; Na^+^ (0), Cl^−^ (10)) were introduced, while preserving a salt concentration of 150 mM. The solvation of the protein was accomplished using the Simple Point Charge (spc216) water model. Using the energy-grps in the MDs parameters (mdp) file, the particle-mesh Ewald method was utilized to study interactions between 5αR2 and eriocitrin, silymarin, and finasteride. To achieve system equilibration, the NPT and NVT ensembles were employed at a temperature of 310 K and pressure of 1 bar. The GROMACS analysis module was utilized to examine trajectories by plotting graphs for Root Mean Square Deviation (RMSD), Root Mean Square Fluctuation (RMSF), Radius of Gyration (Rg), Solvent Accessible Surface Area (SASA), and H-bonding.

## 3. Results and Discussion

5αR2 is involved in the pathophysiology of AGA, and thus, 5αR2 inhibitors are considered crucial for the development of anti-baldness therapies. Accordingly, we screened 241 flavonoids against the active pocket of 5αR2 and identified 11 compounds whose BEs were superior to those of the finasteride (Table 1). These 11 compounds were further evaluated for their interactions with the active site residues of the 5αR2 using Pymol and DS in 2D and 3D view. Through a comprehensive examination of the interactions and thorough investigation of both two-dimensional and three-dimensional interactions, it was determined that eriocitrin and silymarin exhibited the most substantial interaction with 5αR2, as established by visual inspection and interaction analysis.

**Figure 1:**
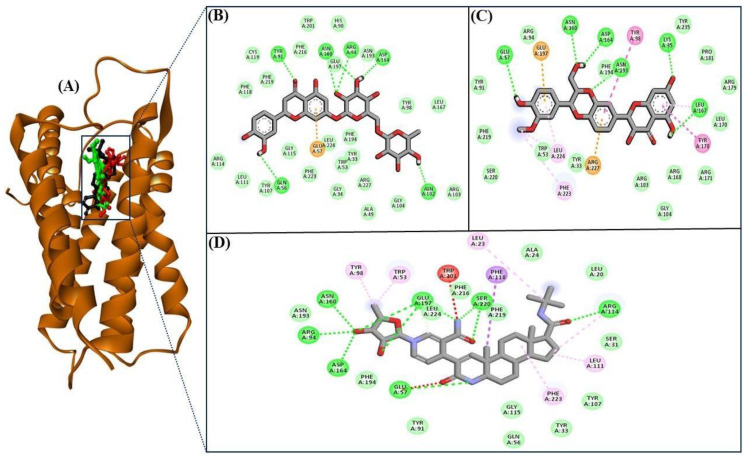
Visualization of finasteride (black), eriocitrin (red), and silymarin (cyan) in the 5αR2 binding pocket (**A**). 2D views of 5αR2 residues interacting with eriocitrin (**B**), silymarin (**C**), and finasteride (**D**).

Figure 1A depicts finasteride, eriocitrin, and silymarin in the 5αR2 binding pocket. The low BE of eriocitrin and silymarin with 5αR2 provided the contributions of H-bonds and van der Waals interactions. Eriocitrin had a BE of −12.1 kcal/mol (Table 1) and interacted with several key residues of 5αR2, viz., Leu11, Tyr33, Gly34, Ala49, Trp53, Gln56, Glu57, His90, Tyr91, Arg94, Tyr98, Asn102, Arg103, Gly104, Tyr107, Arg114, Gly115, Phe118, Cys119, Phe129, Asn160, Asp164, Leu167, Asn193, Phe194, Glu197, Trp201, Phe216, Phe223, Leu224, and Arg227. Of these, the Gln56, Tyr91, Arg94, Asn102, Asn160, and Asp164 residues formed H-bonds with eriocitrin, while Tyr107, Leu111, Arg114, Phe118, Phe219, Cys119, Phe216, Trp201, His90, Glu197, Asn193, Tyr98, Leu167, Arg103, Gly104, Ala49, Arg227, Phe194, Tyr33, Trp53, Gly34, Leu224, Phe223, and Gly115 residues were involved in van der Waals interactions (Figure 1B).

Silymarin had a BE of −11.7 kcal/mol (Table 1) and interacted with the Tyr33, Lys35, Trp53, Glu57, Tyr91, Arg94, Tyr98, Arg103, Gly104, Tyr235, Pro181, Leu167, Asn160, Asp164, Arg168, Leu170, Arg171, Tyr178, Arg179, Asn193, Phe194, Glu197, Phe219, Ser220, Phe223, Leu224, and Arg227 residues of 5αR2. The Lys35, Glu57, Asn160, Asp164, Leu167, and Asn193 residues formed H-bonds with silymarin, while Ser220, Phe219, Tyr91, Arg94, Phe194, Tyr235, Pro181, Arg179, Leu170, Arg171, Arg168, Gly104, Arg103, Tyr33, and Trp53 residues were involved in van der Waals interactions (Figure 1C). The structures of eriocitrin, silymarin, and finasteride are shown in Table 2.

Finasteride, a 5αR2 inhibitor, reduces serum and scalp DHT levels by inhibiting testosterone to DHT conversion and is often used to treat AGA [29]. Clinical studies on men with alopecia revealed that finasteride administration reduced DHT levels in the scalp, promoting hair growth and confirming the role of DHT in the underlying pathophysiology of AGA [30]. In order to obtain a comprehensive understanding of the interacting residues between 5αR2 and the eriocitrin and silymarin, an interaction analysis was conducted on the co-crystallized ligand finasteride (PDB ID: 7BW1) and 5αR2 corresponding residues by redocking the finasteride with 5αR2. The results revealed that the Leu20, Leu23, Ala24, Ser31, Tyr33, Trp53, Gln56, Glu57, Tyr91, Arg94, Tyr98, Tyr107, Leu111, Arg114, Gly115, Phe118, Asn160, Asp164, Asn193, Phe194, Glu197, Trp201, Phe216, Phe219, Ser220, Fhe223, and Leu224 residues of 5αR2 were essential for the interaction with finasteride (Figure 1D). Remarkably, the Trp53, Glu57, Tyr91, Arg94, Tyr98, Asn160, Asp164, Asn193, Phe194, Glu197, Phe223, and Leu224 residues were identified as the common interacting residues of 5αR2 with eriocitrin and silymarin, as well as finasteride (Figure 1B–D), representing that these compounds bind at the same site of 5αR2 as finasteride.

A higher negative BE of a compound with the target enzyme indicates a stronger interaction with its amino acid residues in the catalytic pocket, and the dissociation rate of such compounds from the target enzyme will be slower [31,32,33]. Interestingly, eriocitrin and silymarin had higher (negative) BEs than finasteride (control), revealing that these compounds have a strong interaction with 5αR2.

Silymarin is derived from *Silybum marianum* (L.) *gaernt* (the milk thistle), while *Citrus limon* is a rich source of eriocitrin. The pharmacological effects of these compounds have been well explored, especially their hepatoprotective, antioxidant, anticancer, anti-diabetic, anti-inflammatory, and cardioprotective activities [34,35], and accumulated evidence indicates these compounds are suitable therapeutics. We predicted the physicochemical properties and toxicities of eriocitrin and silymarin (Table 3 and Table 4) and showed that both compounds possess acceptable selected parameters. The physicochemical parameters of the selected compounds were evaluated, including the number of heavy atoms, proportion Csp3, number of rotatable bonds, number of hydrogen bond donors and acceptors, molar refractivity, and TPSA. In addition, the lipophilicity, water solubility, and pharmacokinetics of these compounds were evaluated, and their estimated values are shown in Table 3.

Further, the toxicity assessment of the selected compounds, eriocitrin and silymarin, was assessed by ProTox-II. To estimate a wide range of toxicity endpoints, the ProTox-II methodology employs a comprehensive computational strategy that integrates molecular similarity, pharmacophores, fragment propensities, and machine-learning models. This platform predicts toxicity based on chemical compounds that have been confirmed using various experiments. The web server provides confidence levels for the results and permits similarity comparisons. The predicted lethal dose 50 (pLD50) of eriocitrin was determined to be 12,000 mg/kg, placing it in Toxicity Class six. This classification suggests that eriocitrin exhibits no toxicity. The average similarity between the predicted and actual toxicity statistics for eriocitrin was determined to be 98.6%, while the prediction accuracy was found to be 72.9%. On the other hand, the pLD50 of silymarin was estimated to be 2000 mg/kg, which placed it in the ‘harmful if swallowed’ category and Toxicity Class four. The average similarity between predicted and experimental toxicity was 76.44%, and the prediction accuracy was 69.26%. Toxicity endpoints for silymarin and eriocitrin, such as acute toxicity, hepatotoxicity, cytotoxicity, carcinogenicity, mutagenicity, and immunotoxicity, were within acceptable ranges (Table 4). Furthermore, there was no hepatotoxicity observed in both compounds.

**Figure 2:**
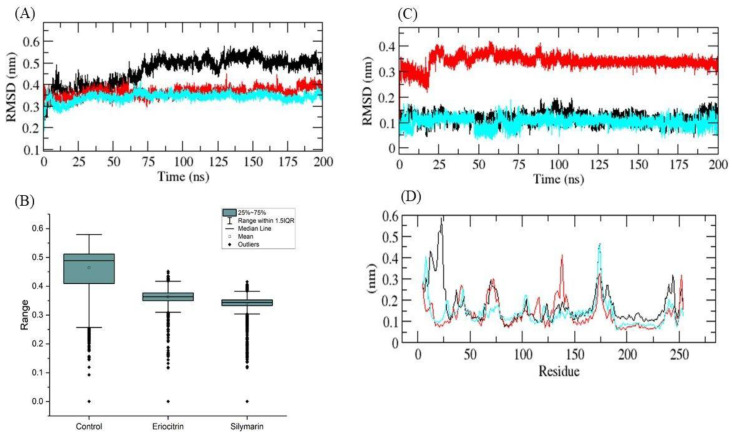
MD simulation studies of docked complexes. RMSD plot (**A**), average RMSD plot (**B**), RMSD plots of ligands in the 5αR2 catalytic pocket (**C**), and corresponding RMSF plots (**D**). Black, red, and cyan indicate finasteride, eriocitrin, and silymarin, respectively.

Subsequently, MD simulations were conducted on the docked complexes of the aforementioned compounds with the 5αR2 enzyme, encompassing a duration of 200 ns. The primary objective of these simulations was to evaluate the stability of the docked complexes. MD simulations primarily aid in the understanding of conformational stability, a phenomenon that has a significant impact on the efficacy of therapeutic compounds in inhibiting target proteins. These simulations, on the other hand, demonstrate their utility by providing insights into interaction dynamics, including bonding events and stability patterns over time. RMSD is a metric utilized to evaluate protein stability, where lower RMSD deviations indicate greater stability. 5αR2-control, 5αR2-eriocitrin, and 5αR2-silymarin had RMSD average values of 0.45, 0.36, and 0.35 nm, respectively, and the RMSD plot revealed that 5αR2-eriocitrin and 5αR2-silymarin complexes had greater binding stability than the 5αR2-control complex. The bound structure of the 5αR2-control complex exhibited high deviation from its initial conformation, whereas the catalytic pocket of 5αR2 formed stable interactions with eriocitrin and silymarin. Furthermore, ligand RMSDs showed that 5αR2-eriocitrin exhibited a high deviation, whereas the 5αR2-control and 5αR2-silymarin complexes exhibited low deviations. It showed that the eriocitrin molecule did not tightly interact in the catalytic pocket of 5αR2 and therefore showed higher deviation in the pocket of the enzyme (Figure 2A–C).

The average fluctuation of all residues throughout the simulation and the RMSD of 5αR2 during the binding of 5αR2-control, 5αR2-eriocitrin, and 5αR2-silymarin was plotted as a function of 5αR2 residue numbers. Consistent fluctuations in the catalytic pocket were observed in the backbones of 5αR2-silymarin and 5αR2-control, possibly due to orientation differences, while the 5αR2-eriocitrin complex exhibited high fluctuation in the 130–140 residue region of 5αR2 (Figure 2D), agreeing with the ligand RMSD which showed high deviation due to high fluctuations in the catalytic pocket of 5αR2. Notably, the 5αR2-silymarin complex demonstrated the least fluctuation overall.

**Figure 3:**
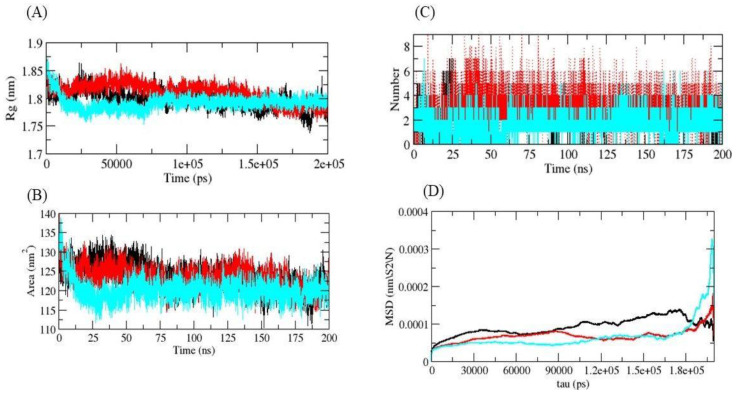
Rg plot of complexes (**A**), SASA plot (**B**), number of H-bonds formed with ligands (**C**), and MSD plot (**D**). Black, red, and cyan indicate finasteride, eriocitrin, and silymarin, respectively.

Rg plots were used to obtain the compactness profiles of the complexes. The 5αR2-control, 5αR2-eriocitrin, and 5αR2-silymarin complexes had average Rg values of 1.79, 1.82, and 1.76 nm, respectively, and the Rg plots showed that 5αR2-eriocitrin and 5αR2-control complexes were less compact than the 5αR2-silymarin complex. These findings suggest that silymarin binding to 5αR2 increased enzyme stability, as evidenced by the reduced Rg compactness and little effect on the 5αR2 structure (Figure 3A), and that silymarin was more stable in the catalytic pocket of 5αR2. On the other hand, eriocitrin induced alteration in the conformational structure of the enzyme, and therefore, less compactness was shown by 5αR2.

SASA provides a measure of the surface area of proteins which interact with solvent molecules. Average SASA values for 5αR2-control, 5αR2-eriocitrin, and 5αR2-silymarin complexes were plotted during the 200 ns simulation, and their SASA values were 128.50, 125.20, and 116.25 nm^2^, respectively (Figure 3B). SASA analysis showed that surface exposure was reduced when silymarin and eriocitrin were bound, while the control compound increased the surface area of solvent accessibility. Thus, 5αR2-eriocitrin and 5αR2-silymarin were found to interact less with the solvent than the 5αR2-control.

**Figure 4:**
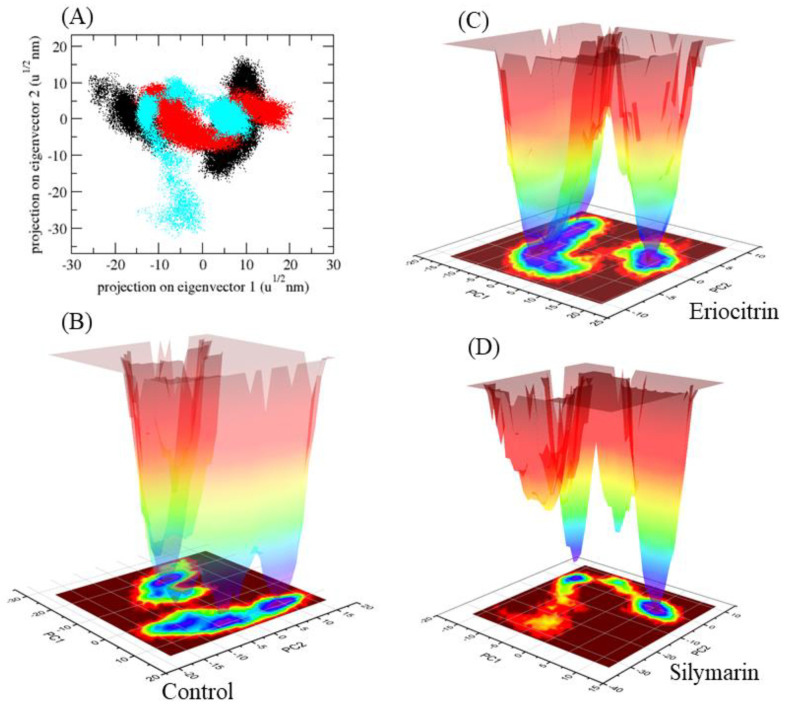
2D projections of complexes (**A**) and Gibbs energy landscape plots (**B**–**D**).

H-bonds represent highly precise interactions between the inhibitor and the target. These interactions are crucial in determining the stability of the complex formed by the target and inhibitor. To evaluate the stabilities of the ligand–target complex, H-bond analysis was conducted during 200 ns simulations of 5αR2-control, 5αR2-eriocitrin, and 5αR2-silymarin in a solvent environment. Finasteride and silymarin were found to form two to four H-bonds with 5αR2, whereas eriocitrin formed two to six H-bonds (Figure 3C). In addition, the mean square displacement (MSD) of atoms from a set of original 5αR2 complex coordinates was computed (Figure 3D). The displacement of atoms from a set of beginning sites in the complexes 5αR2-control, 5αR2-eriocitrin, and 5αR2-silymarin was calculated, with 5αR2-silymarin having the greatest MSD value. The first two eigenvectors were projected in 2D. During the simulations, the 5αR2-control and 5αR2-eriocitrin exhibited a decreased diversity of conformation during simulation; however, the 5αR2-silymarin complex exhibited a greater diversity of conformations. This demonstrates that the 5αR2-silymarin complex was efficiently equilibrated and stable during the simulation (Figure 4A).

Further, GROMACS analysis modules were used to calculate the Gibbs’ free energy (GFE) landscape and project the respective first (PC1) and second (PC2) eigenvectors (darker blue shades indicate lower energy levels). During the simulations, ligand binding to 5αR2 caused fluctuations in the global minima of 5αR2, as observed in the GFE contour maps. 5αR2-control and 5αR2-silymarin had similar projections, whereas 5αR2-eriocitrin had different global minima, indicating that the global minima of eriocitrin had changed during simulation (Figure 4B–D).

Over the years, various potential 5αR2 inhibitors have been explored, and some have been synthesized. Furthermore, potent 5αR2 inhibitors have consistently been found to bind strongly to 5αR2 [36]. Finasteride and dutasteride are currently being used to treat AGA [37,38], but both have been associated with adverse effects like impotency and sexual dysfunction [38,39]. Thus, there is an urgent need to identify natural 5αR2 inhibitors with no side effects. Natural products and traditional medicines are extremely important, and their derivatives have long been recognized as valuable reservoirs of therapeutic agents and structural variability. A wide range of pharmaceutical agents currently on the market have their origins in natural reservoirs. Natural products have long been used to identify potential developmental leads [40,41,42,43,44], and in this study, we found that natural flavonoids, namely, eriocitrin and silymarin, stably interact with 5αR2, which implies their potential as therapeutic agents for treating AGA.

## 4. Conclusions

The involvement of 5αR2 in the pathophysiological mechanisms of AGA establishes the necessity of 5αR2 inhibitors in the advancement of therapeutic interventions for AGA. VS has been routinely employed to identify new drug leads. In the present study, in silico methodologies, viz., molecular docking-based VS, toxicity prediction, and MD simulation, were used to identify potential nontoxic 5αR2 inhibitors in a natural flavonoid library. Eriocitrin and silymarin were found to interact strongly with 5αR2 and form stable complexes and could be potential future anti-baldness drug candidates. However, further experimental research is needed to optimize them as 5αR2 inhibitors.

## Figures and Tables

**Table 1:** BE of top-screened flavonoid compounds.

S. No.	Compound Name	Binding Energy (kcal/mol)
1.	Eriocitrin	−12.1
2.	Obacunone	−11.8
3.	Oroxin_B	−11.7
4.	Silymarin	−11.7
5.	Hesperidin	−11.7
6.	Baicalin	−11.6
7.	Diosmin	−11.6
8.	Scutellarin	−11.6
9.	Methyl-Hesperidin	−11.6
10.	Narirutin	−11.6
11.	Isosilybin	−11.6
12.	Finasteride (positive control)	−11.2

**Table 2:** Structures of eriocitrin, silymarin, and finasteride and interacting residues of 5αR2.

Compounds	Structure	No. of H-Bond	H-Bonds Interacting Residues	Van der Waals Interactions
Eriocitrin	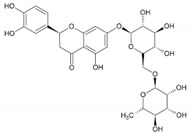	6	Gln56, Tyr91, Arg94, Asn102, Asn160, and Asp164	Tyr107, Leu111, Arg114, Phe118, Phe219, Cys119, Phe216, Trp201, His90, Glu197, Asn193, Tyr98, Leu167, Arg103, Gly104, Ala49, Arg227, Phe194, Tyr33, Trp53, Gly34, Leu224, Phe223, and Gly115
Silymarin	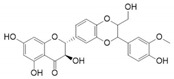	6	Lys35, Glu57, Asn160, Asp164, Leu167, and Asn193	Ser220, Phe219, Tyr91, Arg94, Phe194, Tyr235, Pro181, Arg179, Leu170, Arg171, Arg168, Gly104, Arg103, Tyr33, and Trp53
Finasteride	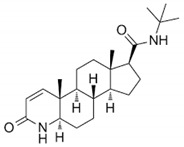	7	Glu57, Arg94, Arg114, Asn160, Asp164, Glu197, and Ser220	Asn193, Leu224, Tyr91, Phe194, Phe216, Phe219, Ala24, Leu20, Ser31, Tyr107, Tyr33, Gly115, and Gln56

**Table 3:** Estimated physicochemical properties of silymarin, eriocitrin, and finasteride.

Properties		Compound Name		
Physicochemical Properties		Eriocitrin	Silymarin	Finasteride (Control)
MW		596.53	482.44	372.54
Heavy atoms		42	35	27
Aromatic heavy atoms		12	18	0
Fraction Csp3		0.52	0.24	0.83
RB		6	4	3
HBA		15	10	2
HBD		9	5	2
Molar Refractivity		136.94	120.55	113.18
TPSA		245.29	155.14	58.20
Lipophilicity				
iLOGP		1.95	2.79	3.32
XLOGP3		−1.35	1.9	3.03
WLOGP		−1.78	1.71	3.43
MLOGP		−3.24	−0.4	3.46
Silicos-IT Log P		−2.1	1.92	3.20
Consensus Log P		−1.3	1.59	3.29
Water Solubility				
ESOL	Log S	−2.5	−4.14	−3.86
	Solubility (mg/mL)	1.87 × 10^0^	3.46 × 10^−2^	5.13 × 10^−2^
	Class	Soluble	Moderately soluble	Soluble
Ali	Log S	−3.3	−4.78	−3.92
	Solubility (mg/mL)	2.98 × 10^−1^	7.99 × 10^−3^	4.50 × 10^−2^
	Class	Soluble	Moderately soluble	Soluble
Silicos-IT	LogSw	0.1	−4.5	−4.54
	Solubility (mg/mL)	7.56 × 10^2^	1.53 × 10^−2^	1.07 × 10^−2^
	class	Soluble	Moderately soluble	Moderately soluble
Pharmacokinetics				
GI absorption		Low	Low	High
BBB permeant		No	No	Yes
Pgp substrate		Yes	No	Yes
inhibitor	CYP1A2	No	No	No
	CYP2C19	No	No	No
	CYP2C9	No	No	No
	CYP2D6	No	No	No
	CYP3A4	No	Yes	No
log Kp (cm/s)		−10.9	−7.89	−6.42

**Table 4:** Toxicity predictions for silymarin, eriocitrin, and finasteride.

Classification	Target	Prediction			Probability		
		Silymarin	Eriocitrin	Finasteride (Control)	Silymarin	Eriocitrin	Finasteride (Control)
Organ toxicity	Hepatotoxicity	IA	IA	IA	0.78	0.8	0.98
Toxicity endpoints	Carcinogenicity	IA	IA	IA	0.72	0.91	0.61
	Immunotoxicity	A	A	A	0.97	0.99	0.99
	Mutagenicity	IA	IA	IA	0.69	0.88	0.81
	Cytotoxicity	IA	IA	IA	0.77	0.64	0.79
Tox21-Nuclear receptor signaling pathways	AhR	A	IA	IA	0.99	0.83	0.99
	Androgen Receptor (AR)	IA	IA	IA	0.95	0.98	0.87
	AR-LBD	IA	IA	IA	0.99	0.99	0.99
	Aromatase	IA	IA	IA	0.8	0.99	0.97
	Estrogen Receptor Alpha (ER)	IA	IA	IA	0.71	0.95	0.93
	ER-LBD	IA	IA	IA	0.96	0.99	0.98
	PPAR-Gamma	IA	IA	IA	0.97	0.98	0.98
Tox21-stress response pathways	nrf2/ARE	IA	IA	IA	0.92	0.99	0.97
	HSE	IA	IA	IA	0.92	0.99	0.97
	MMP	IA	IA	IA	0.73	0.97	0.93
	p53	IA	IA	IA	0.91	0.9	0.97
	ATAD5	IA	IA	IA	0.94	0.99	0.99

(IA = Inactive; A = Active).

## Data Availability

Not applicable.
